# A user-friendly, low-cost turbidostat with versatile growth rate estimation based on an extended Kalman filter

**DOI:** 10.1371/journal.pone.0181923

**Published:** 2017-07-26

**Authors:** Stefan A. Hoffmann, Christian Wohltat, Kristian M. Müller, Katja M. Arndt

**Affiliations:** 1 Molecular Biotechnology, Institute for Biochemistry and Biology, University of Potsdam, Potsdam-Golm, Germany; 2 Cellular and Molecular Biotechnology, Faculty of Technology, Bielefeld University, Bielefeld, Germany; The Ohio State University, UNITED STATES

## Abstract

For various experimental applications, microbial cultures at defined, constant densities are highly advantageous over simple batch cultures. Due to high costs, however, devices for continuous culture at freely defined densities still experience limited use. We have developed a small-scale turbidostat for research purposes, which is manufactured from inexpensive components and 3D printed parts. A high degree of spatial system integration and a graphical user interface provide user-friendly operability. The used optical density feedback control allows for constant continuous culture at a wide range of densities and offers to vary culture volume and dilution rates without additional parametrization. Further, a recursive algorithm for on-line growth rate estimation has been implemented. The employed Kalman filtering approach based on a very general state model retains the flexibility of the used control type and can be easily adapted to other bioreactor designs. Within several minutes it can converge to robust, accurate growth rate estimates. This is particularly useful for directed evolution experiments or studies on metabolic challenges, as it allows direct monitoring of the population fitness.

## Introduction

During growth of microorganisms in batch cultures, complex and dynamic environmental changes take place, which have a considerable impact on cell physiology [1–5]. Those physiological alterations are often undesired for experimentation. For instance, the dynamically changing environment of batch cultures can introduce unwanted selective pressure in evolutionary experiments [6]. To achieve continuous, constant culture conditions, two different general approaches are commonly employed. The two corresponding bioreactor types are called chemostats and turbidostats. In chemostats, culture medium is continuously exchanged at a fixed dilution rate. Due to nutrient depletion or toxin accumulation growth will eventually be restricted by the supply of fresh medium and thus create a constant steady-state environment. Such devices can have a simple design [7], but their use is virtually limited to applications in which metabolic restriction of growth rates is desired. In contrast, in turbidostats a specified cell density is held constant by medium exchange on demand. This requires a feedback control, making technical solutions more demanding. However, turbidostats enable researchers to create constant environmental conditions at adjustable equilibrium cell densities. This is e.g. particularly useful in directed evolution experiments [8–11], for investigating the reaction of microorganisms to metabolic stimuli [12,13], or for characterization of engineered biological circuitry [14]. However, currently commercially available turbidostats are not very widespread due to considerable cost of procurement and maintenance, especially if parallelization of experiments is desired. Researchers therefore have come up with different custom-built designs [10,11,14–16].

We have developed a turbidostat design with emphasis on ease of operation and manufacture as well as low costs, which features a novel growth rate estimation algorithm. With the employed Kalman filter-based approach, information about growth rates is not reset at dilution events, which makes its estimates substantially more robust. Yet, the algorithm readily tracks growth rate dynamics, allowing real-time assessment of population fitness. Furthermore, like the ON/OFF feedback control used here for density control, the growth rate estimation algorithm does not require parametrization of culture volume or exact dilution rates. Due to its general approach, it can easily be adapted for other bioreactor designs with discrete dilution events by adjustment of the parameter describing the measurement variance of the used optical system.

## Results and discussion

### System description

**Fig 1:**
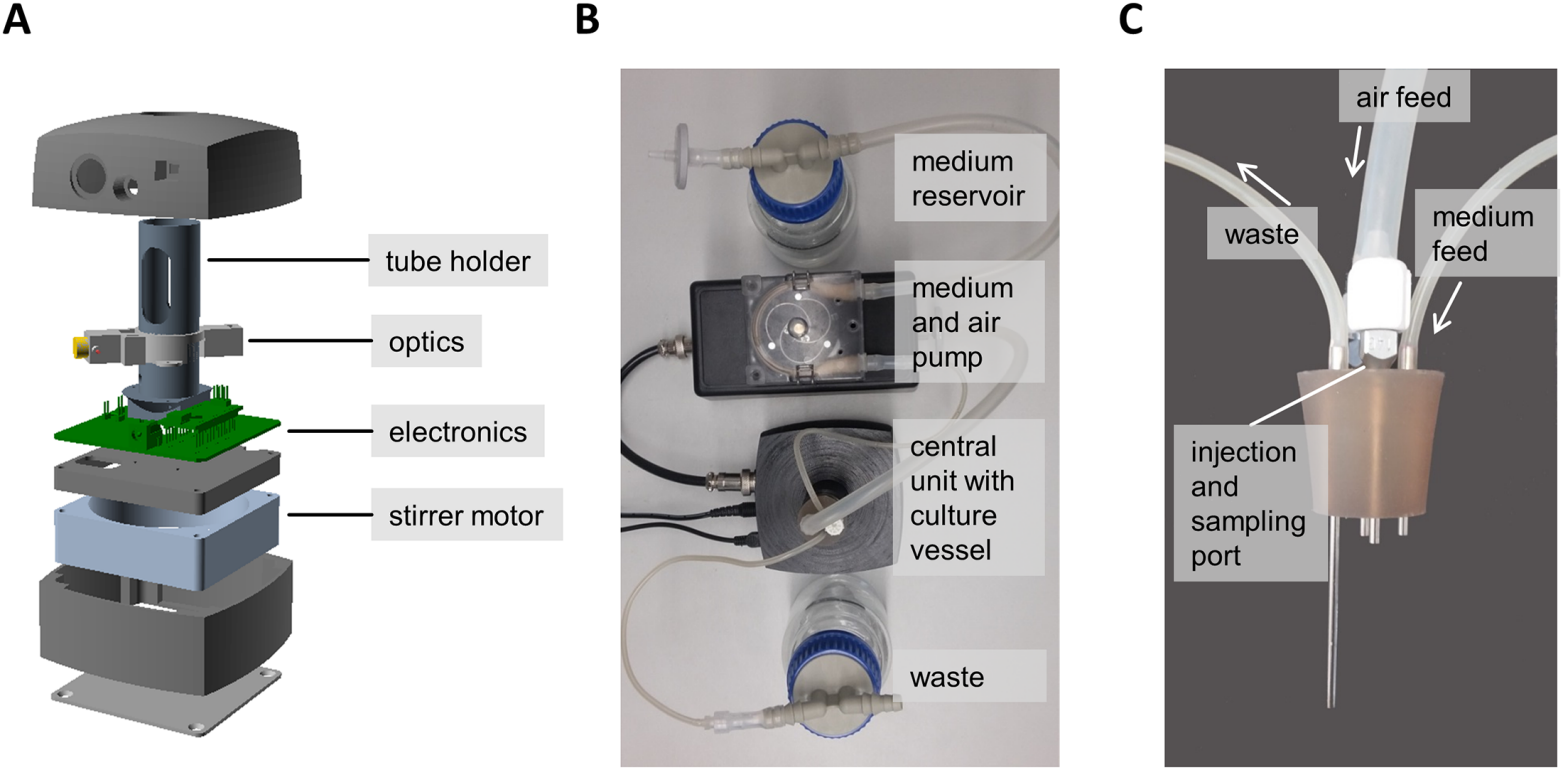
Turbidostat overview. (A) Explosion model of the central unit, showing 3D printed parts in black or grey. Optics consist of a 650 nm laser module, a beam splitter and two light-to-frequency converters, integrated into an optics holder mounted onto the tube holder. The stirrer motor is a standard 80 mm computer fan with a bar magnet attached to the rotor. (B) Photograph of the assembled turbidostat system (top view). (C) Photograph of the culture vessel’s silicone plug with four ports.

**Fig 2:**
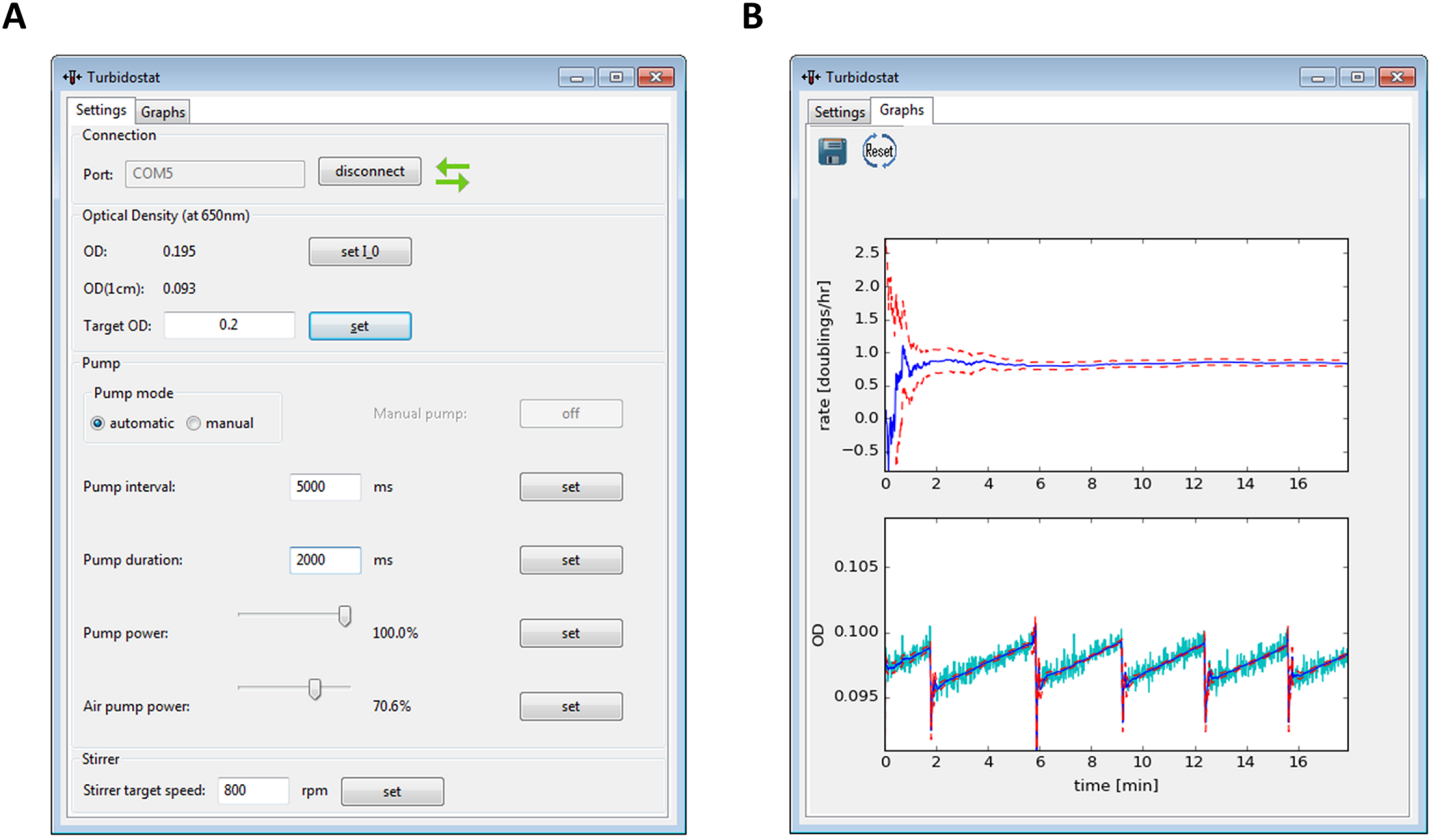
Graphical user interface. (A) Settings tab for turbidostat operation. (B) Graphs tab for on-line display of estimated doubling rates with estimate standard deviation (upper panel) and measured and estimated optical densities (lower panel). The implemented time series analysis algorithm jointly estimates the actual, underlying optical densities and the doubling rates. Measured ODs are shown in cyan, parameter estimates are shown in blue and associated estimation errors as dashed, red lines. After algorithm initialization, initial convergence to a good estimate occurs within few minutes. Within the shown timeframe, five dilution events occurred. The triggered increases in OD estimate standard deviation and subsequent rapid estimate re-convergence can be seen.

Using 3D printing and programmable microcontrollers we have developed a small-scale, user-friendly turbidostat that can be built for under 300 € of material cost. We opted to achieve a high degree of spatial system integration (Fig 1) and implemented an intuitive graphical user interface to allow easy operation (Fig 2). The culture chamber, sensory and control electronics are integrated into a central unit, which was manufactured with a fused deposition modeling 3D printer (Fig 1A). A peristaltic pump for culture medium and an air pump are built into a separate, generic housing (Fig 1B). The culture volume of the culture chamber, a flat-bottom test tube with magnetic stir bar for agitation, is variable and regulated by the position of the effluent tube, and is aerated by pressurized sterile air from the air pump. The air pressure drives out medium above the level of the opening of the effluent tube (Fig 1C). For temperature control the device is placed in an incubator. The optical set-up for optical density (OD) measurement is similar to that of a previously reported turbidostat design [14], and its measured densities of bacterial cultures show near-perfect linearity with the measurements of a commercial photometer up to a density of 1.5 (S1 Fig). In short, the beam of a 650 nm laser diode is split before going through the culture chamber, with most of the light going to the culture chamber, being detected by a light-to-frequency converter on the other side. The part of the beam not going through the culture chamber is detected by a second light sensor for signal correction by using the ratio between the two signals to calculate optical densities.

The basic functionality is encoded on an Arduino Nano microcontroller. For regulation of the optical density, a simple ON/OFF feedback control is used: The optical densities are measured every second and compared to the target density. As soon as the target OD is surpassed, a pump event is triggered, diluting the culture. The medium pump is turned on for the duration specified in the program’s settings tab (“pump duration”). Between pump events a minimum delay can be set (“pump interval”). Here, minimum pump intervals of 5 seconds were chosen with pump durations of 2 seconds at a pump power of 100% (12 V). With the used peristaltic pump, this equals approximately 800 μl per pump event on average, exchanging roughly 4% of the culture volume, which was 20 ml in all experiments. For user-friendly parameter operation, a Python based graphical user interface (GUI) has been implemented, which communicates with the microcontroller, calculates growth rates, and creates log files. It allows easy setting of all operation parameters and it plots measured and estimated optical density as well as estimated doubling rates in real time (Fig 2).

### Growth rate estimation by extended Kalman filter

To allow robust on-line growth rate estimation while retaining the flexibility associated with the used ON/OFF feedback control, an extended Kalman filter [17] has been adapted and implemented into the graphical user interface. The Kalman filter is an iterative Bayesian estimation algorithm useful to estimate system states from sequential, noisy measurements. From a currently estimated state, the state in the next time step is predicted by a state transition model, which describes the theory of the dynamic system behavior. The information of the prediction is then combined with the information of the measurement, each weighed with its respective uncertainty. This combination yields an estimate with reduced uncertainty, which in turn is used for the prediction of the following state. The original filter deals with linear dynamic systems, but one method for its application to non-linear systems is based on linearization at each time step by differentiation. The resulting filter is called an extended Kalman filter.

An extended Kalman filter has been applied to on-line estimation of growth rates as follows. At a given time point *k* the state *x*_*k*_ of the culture is defined by the two components optical density OD and a growth rate *r*:

$$x_{k}=\left[\begin{array}{c} OD_{k}\\ r_{k} \end{array}\right]$$ (1)

For the OD transitions from one time point to the next, a geometric growth model is used as recursive, discrete equivalent of an assumed exponential growth:

$$OD_{k+1}=OD_{k}\;*\;r_{k}$$ (2)

This yields a non-linear system; the function *f* is used to predict the state in *k*+1 from the state estimate for the previous time step ${\hat{x}}_{k|k}$:

$${\hat{x}}_{k+1|k}=f\left({\hat{x}}_{k|k}\right)=\;\left[\begin{array}{c} {\hat{OD}}_{k|k}\text{ * }{\hat{r}}_{k|k}\\ {\hat{r}}_{k|k} \end{array}\right]$$ (3)

The pre-measurement state estimate ${\hat{x}}_{k+1|k}$ and its associated covariance is then used as prior probability distribution for a Bayesian parameter estimation performed with the new information from the measurement in *k*+1. This yields the post-measurement state estimate ${\hat{x}}_{k+1|k+1}$, which in turn is used for state prediction of the following step. Thus, this recursive algorithm implicitly incorporates past cell density measurements and the estimate variances decrease with multiple consecutive observations. Importantly, the underlying model does not require information about the culture volume or dilution rates, retaining the user’s high degree of flexibility concerning the choice of associated parameters. Measurements and updates are performed once a second. For display, estimated geometric growth rates and associated estimation errors are transformed to doubling rates per hour.

In turbidostat operation, the system is recurrently disturbed by dilution events triggered by OD set point transgression. To account for those disturbances, the OD variance component is assigned a high estimate uncertainty during a dilution event and the following ten measurement cycles. Due to this partial reset of the estimate covariance matrix, new measurements are attributed a high weight for OD estimation, leading to a quick reconvergence to the altered density. The other covariance matrix elements remain unchanged, maintaining the information on the growth rate estimate and attributing a low weight to new measurements, until the OD estimation has reconverged.

Dilution rates should be chosen high enough, such that there are roughly 100 or more measurement cycles (i.e. seconds) between two subsequent dilution events on average. Thus, a reasonable ratio between “meaningful” measurements and “distrusted” measurements is achieved, allowing the algorithm to converge to good estimates despite dilution events. A video clip of the state estimator is provided in S4 File.

### Simulations

Performances of the presented extended Kalman filter based growth rate estimation and the often-applied conventional method using simple linear regression of logarithmized optical densities between dilution events, were compared using simulated data with known growth rates.

**Fig 3:**
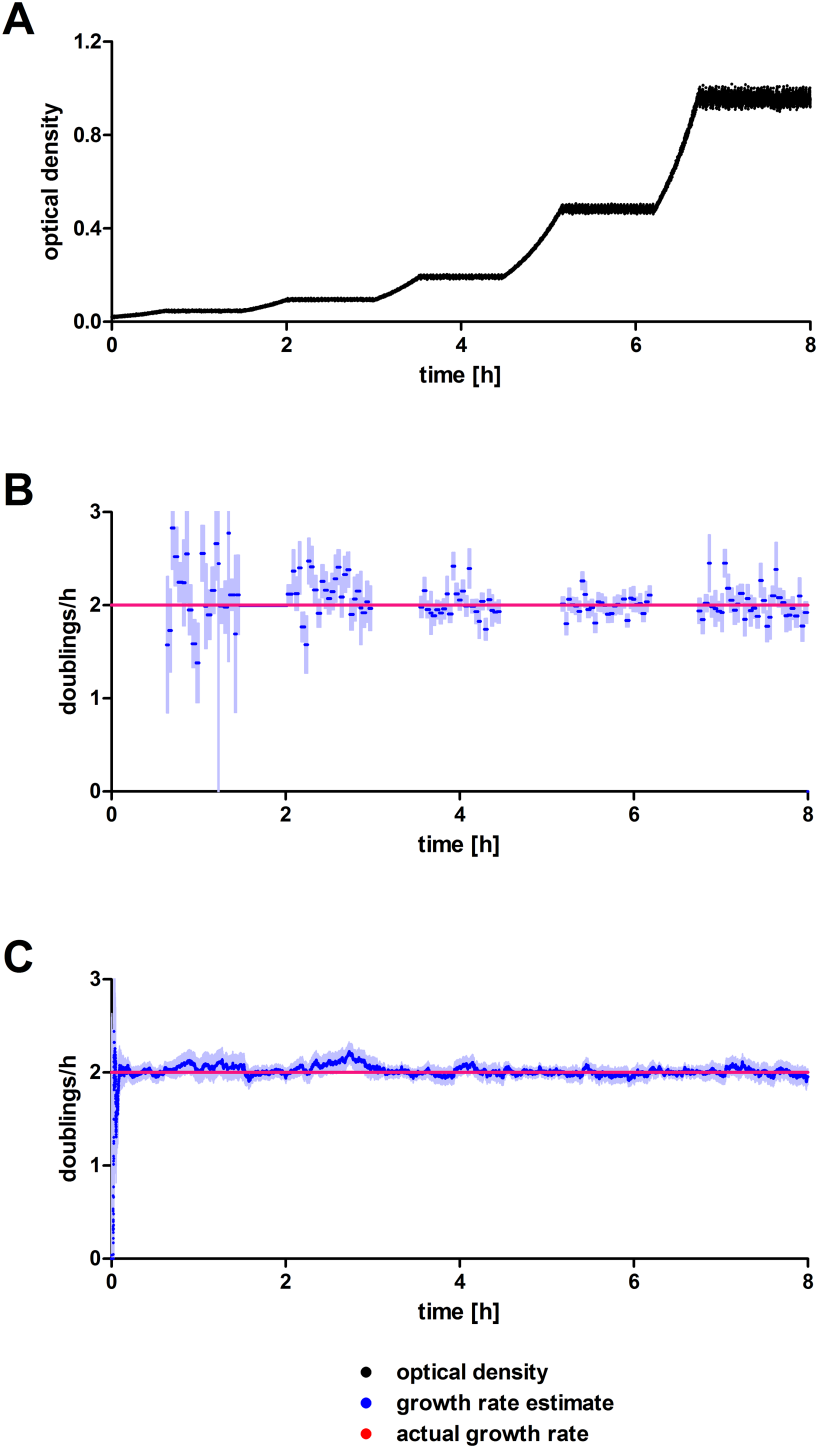
Simulated turbidostat run with incrementing target densities (0.05, 0.1, 0.2, 0.5, 1) and a constant growth rate of 2 doublings per hour. (A) Simulated density measurements. (B) Growth rate estimates by simple linear regression of logarithmized densities between dilution events. (C) Growth rate estimates by Kalman filter. Point estimates are shown in lush blue and the estimates’ standard errors in faint blue. The actual doubling rate is shown in red in (B) and (C).

To assess the performance of both estimation algorithms at a range of different optical densities, a course with incrementing target densities was simulated including measurement noise (Fig 3). Actual growth rates were held constant at 2 doublings per hour. At low target densities the conventional approach yields strongly fluctuating estimates with high uncertainties because the absolute change of intensities and hence the signal-to-noise ratio within one dilution cycle is lower than at higher optical densities. In contrast, the Kalman filter-based estimates are stable over the whole range of densities.

**Fig 4:**
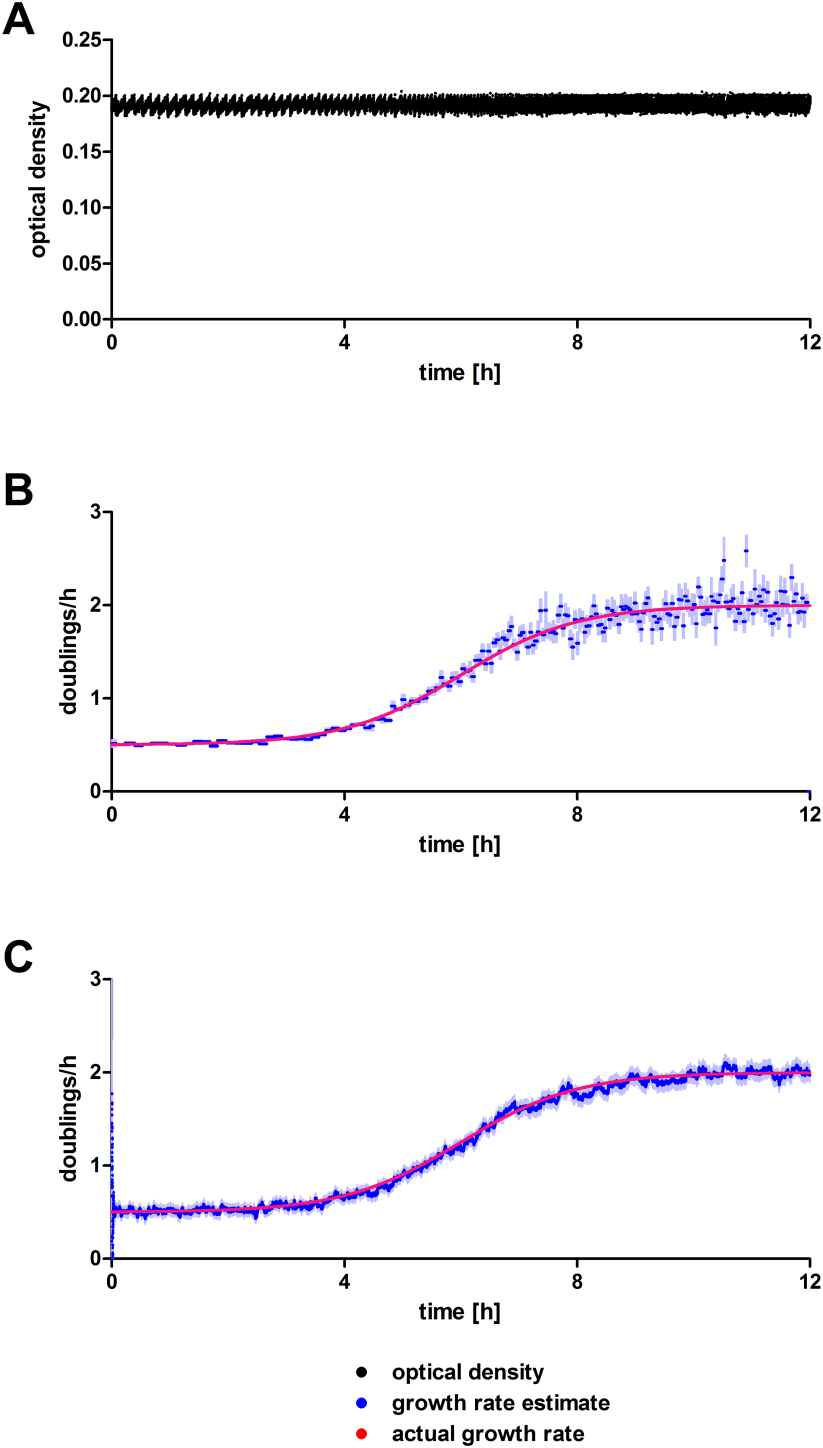
Simulated turbidostat run with sigmoidally increasing doubling rate. (A) Simulated density measurements. (B) Growth rate estimates by simple linear regression of logarithmized densities between dilution events. (C) Growth rate estimates by Kalman filter. Point estimates are shown in lush blue and the estimates’ standard errors in faint blue. The actual doubling rate is shown in red in (B) and (C).

Further, both algorithms were compared in a situation with varying growth rates at a constant density of 0.2 (Fig 4). The doubling rates were increased from 0.5 doublings per hour to 2 doublings per hour in a sigmoidal fashion. This sigmoidal transition would be expected e.g. in selection experiments, which enrich high fitness phenotypes with initially low frequencies from a background of low fitness phenotypes. The regression-based method yields good estimates when growth is slow. However, with increasing growth rates its estimates become more unreliable because dilution cycles are shorter and thus fewer data points are available between two dilution events. The Kalman filter-based estimates have slightly increased estimate uncertainties at higher rates as well, but estimates are appreciably more robust.

### Continuous culture at different densities

**Fig 5:**
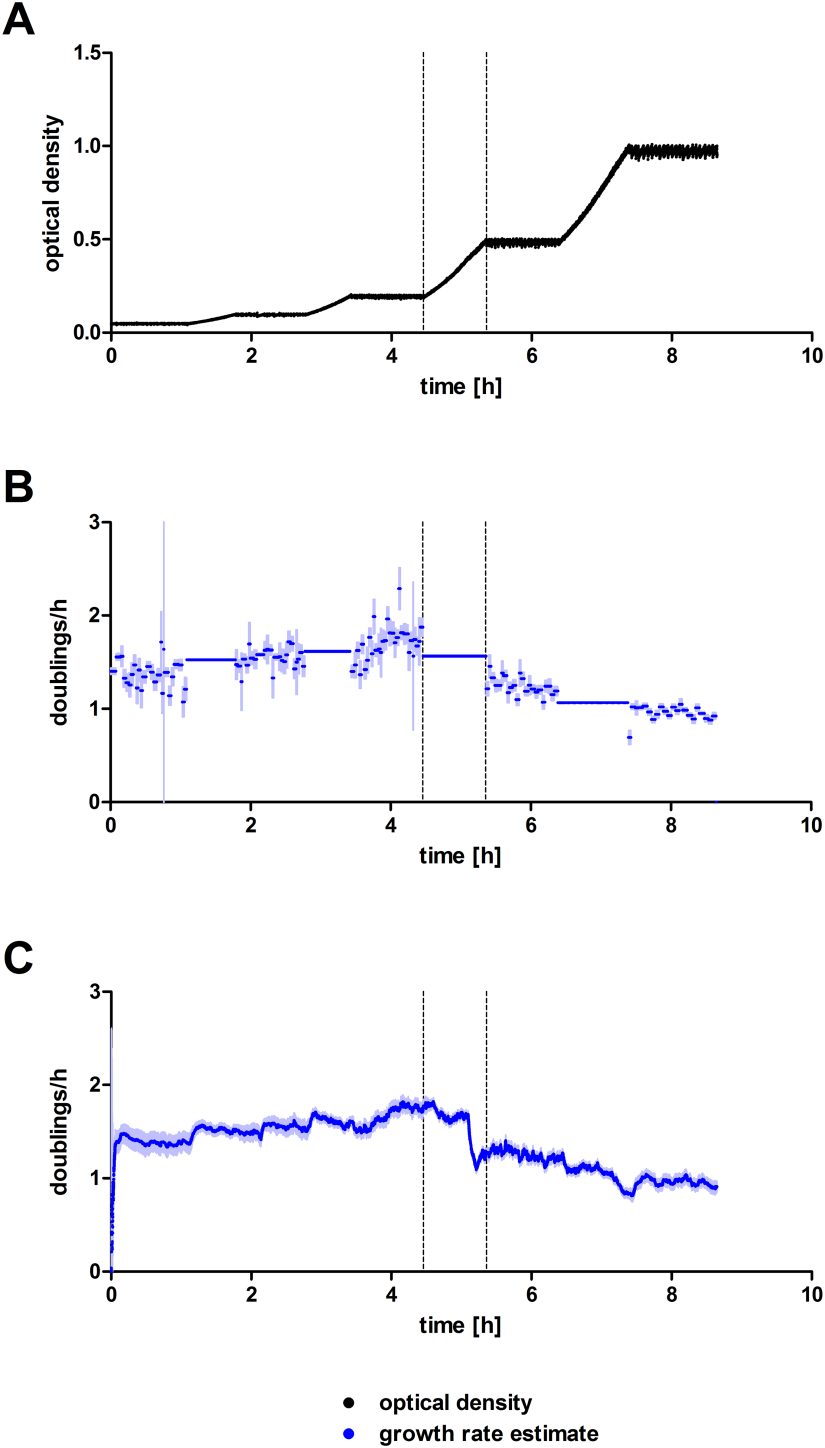
Continuous culture of *E*. *coli* BL21 with incrementing target densities (0.05, 0.1, 0.2, 0.5, 1). (A) Measured optical densities. (B) Doubling rates calculated by linear regression of logarithmized optical densities and (C) doubling rate estimates from the extended Kalman filter. Point estimates are shown in lush blue and the estimates’ standard errors in faint blue. The transition from a target density of 0.2 to 0.5 is marked with dashed lines.

*E*. *coli* BL21 were cultivated in the turbidostat with incrementing target densities (0.05, 0.1, 0.2, 0.5, 1). The used ON/OFF feedback control evidently allows continuous culture at a wide range of optical densities with almost the same average relative difference between target and actual densities (Fig 5A). The doubling rates estimated by the implemented extended Kalman filter were compared to doubling rates, which were calculated by simple linear regression of the log_2_ transformed optical densities between two subsequent pump strokes (Fig 5B and 5C). The estimates are generally in good agreement. However, during constant density phases with frequent interruptions by pump events, regressions between two pump events yielded strongly fluctuating estimates with high uncertainties, whereas the Kalman filter’s estimates were notably more stable. During the transition periods from one OD plateau to the next, the linear regression naturally does not resolve growth rate dynamics within the associated time frames, giving an average value instead. Here, the Kalman filter algorithm readily detected changes in doubling rates, e.g. the drop in speed of growth during the transition from OD 0.2 to 0.5.

### Competition of *E*. *coli* strains

**Fig 6:**
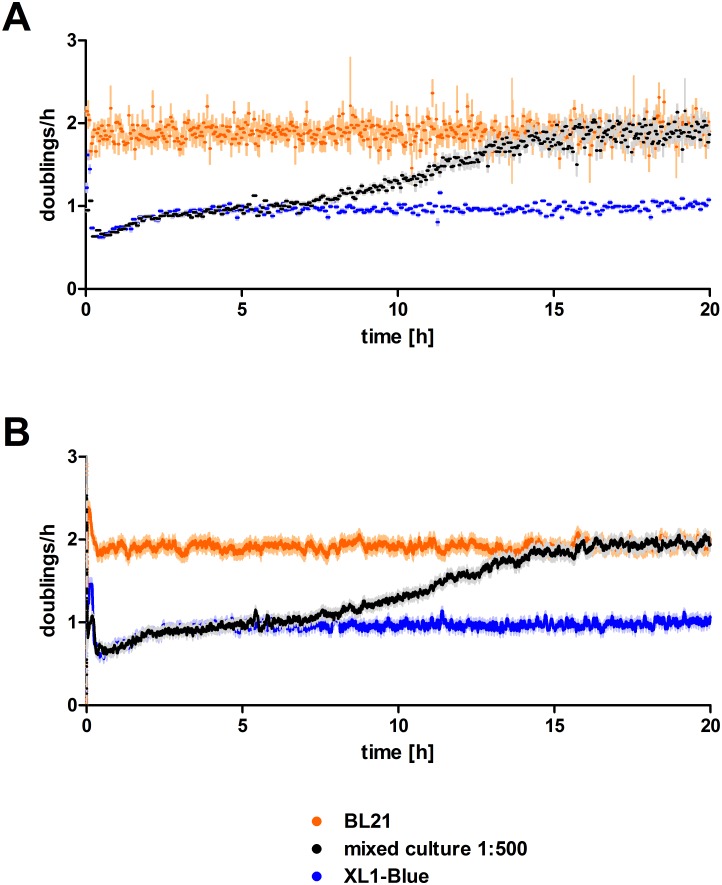
Pure and mixed cultures of *E*. *coli* strains XL1-Blue and BL21 with growth rate estimation by (A) regression-based and (B) Kalman filter-based approach. Estimates for the pure culture of BL21 are shown in orange, for XL1-Blue in blue. The mixed culture, starting with a 500-fold excess of XL1-Blue with regard to optical density, is shown in black. Estimation standard errors are shown in the respective faint color.

As an experimental demonstration of a competition of two genotypes with different fitness, the *E*. *coli* strains XL1-Blue and BL21 were grown in the turbidostat separately and as mixed cultures (Fig 6). In supplemented M9 medium they displayed a pronounced difference in growth speed. Both algorithms, the regression based conventional approach and the presented Kalman filter, allowed to track the fast growing strain BL21 outcompeting the slow growing strain XL1-Blue. Mixed cultures with an initial 500-fold excess of XL1-Blue initially behaved as a pure XL1-Blue culture, and subsequently showed a sigmoidal increase in growth rate reaching that of BL21. As seen in the simulation with increasing growth rates, estimates of the regression-based method tend to strongly fluctuate at higher growth rates (Fig 4). While those estimates can be useful for analysis after the actual experiment has been finished (e.g. by fitting), on-line assessments of the current population fitness proved difficult. In contrast, the Kalman filter based approach yielded stable estimates for the entire range of covered doubling rates, providing a substantially better on-line estimation of the actual underlying growth rate. Thus, an increasing adaptation of the population can be monitored in real time, e.g. helping to avoid over-selection in directed evolution studies.

## Material and methods

### Materials

A list of used commercially available parts is given in S1 Table. Individually printed parts and resulting assembly groups are shown in S2 Fig, and files for printing are given in S1 File. The board scheme is shown in S3 Fig. The board layout for reproduction is given in S2 File.

### State estimator

A generic extended Kalman filter algorithm [18] was adapted for on-line estimation of doubling rates as follows. The state transitions of the state components are given as geometric, discrete growth model with the optical density *OD* and the geometric growth rate *r* as state components:

$$OD_{k+1}=OD_{k}\;*\;r_{k}$$ (4)

$$r_{k+1}=r_{k}$$ (5)

The combined function *f* is used to predict the state ${\hat{x}}_{k+1|k}$ in *k+1* from the previous state estimate ${\hat{x}}_{k|k}$ already corrected with the measurement in *k*, yielding a non-linear system:

$${\hat{x}}_{k+1|k}=f\left({\hat{x}}_{k|k}\right)=\;\left[\begin{array}{c} {\hat{OD}}_{k|k}\text{ * }{\hat{r}}_{k|k}\\ {\hat{r}}_{k|k} \end{array}\right]$$ (6)

The projection of the estimation covariance to the subsequent time step is done by:

$$P_{k+1|k}=F_{k}P_{k|k}F_{k}^{T}+Q$$ (7)

Here, *P* is the estimation error, *F* the Jacobian matrix of *f* Eq (8), and *Q* is the process noise covariance.

$$F_{k}=\;{\frac{\partial f}{\partial x}|}_{{\hat{x}}_{k|k}}=\left[\begin{array}{cc} {\hat{r}}_{k|k} & {\hat{OD}}_{k|k}\\ 0 & 1 \end{array}\right]$$ (8)

The observable read-out of the system is a ratio of the forward and reference light intensity as measured by the two light-to-frequency converters, and is here simply referred to as (corrected) light intensity *I*. To compute optical densities, a blank intensity measurement with medium but without cells has to be performed, yielding *I*_*blank*_. The light intensity *I* at the time step *k* can be written as a function of the optical density *OD*, which is a component of the system state:

$$I_{k}=h\left(OD_{k}\right)=\;\frac{I_{blank}}{10^{OD_{k}}}$$ (9)

The observation model thus is defined as:

$$z_{k}=\;h\left(OD_{k}\right)+v_{k}$$ (10)

where *v*_*k*_ is the measurement noise. After each measurement the state estimate and the estimation covariance are updated, incorporating the new information:

$${\hat{x}}_{k+1|k+1}=\;{\hat{x}}_{k+1|k}+\;K_{k+1}\left[z_{k+1}-h\left({\hat{x}}_{k+1|k}\right)\right]$$ (11)

$$P_{k+1|k+1}=\left(I-K_{k+1}H_{k+1}\right)P_{k+1|k}$$ (12)

The Kalman gain *K*_*k*+1_ is computed as follows:

$$K_{k+1}=\;P_{k+1|k}H_{k+1}^{T}{\left(H_{k+1}P_{k+1|k}H_{k+1}^{T}+R\right)}^{-1}$$ (13)

*H* is the Jacobian matrix of the measurement conversion function *h* Eq (14).

$$H_{k+1}={\frac{\partial h}{\partial x}|}_{{\hat{x}}_{k+1|k}}=\left[\begin{array}{cc} -\frac{\text{ln}\left(10\right)\;*\;I}{10^{{\hat{OD}}_{k+1|k}}} & 0 \end{array}\right]$$ (14)

The measurement noise covariance *R*, the process noise covariance *Q*, and the initial estimate error *P*_*0*_ were parameterized as follows:

$$R\;=\text{var}(v_{k})=\;40^{2}$$ (15)

$$Q=\;\left[\begin{array}{cc} q_{11} & q_{12}\\ q_{21} & q_{22} \end{array}\right]=\left[\begin{array}{cc} 0 & 0\\ 0 & 5\;*\;10^{-13} \end{array}\right]$$ (16)

$$x_{0}=\left[\begin{array}{c} OD_{0}\\ 1 \end{array}\right]$$ (17)

$$P_{0}=\;\left[\begin{array}{cc} p_{11,0} & p_{12,0}\\ p_{21,0} & p_{22,0} \end{array}\right]=\left[\begin{array}{cc} R/{\left(I_{0}\;*\;ln10\right)}^{2} & 0\\ 0 & 0.0005^{2} \end{array}\right]$$ (18)

The measurement variance was first assessed by determining variances of measured intensities at different optical densities (between 0.02 and 1) of methylene blue. Measurement variance determined this way (below 20^2^) appeared to underestimate the variance observed when growing bacterial cells. Therefore, *E*. *coli* BL21 growth curves were recorded in the turbidostat, and between an OD of 0.1 and 0.2 the growth rate appeared to be constant. For the respective data points, densities were log transformed and a linear regression was performed. Variance of the data to the regression line was between 31^2^ and 33^2^. For parametrization of the Kalman filter a slightly higher measurement variance of 40^2^ was chosen to account for a possibly higher variance at higher cellular densities or with different microorganisms.

The process variance for the state component ‘optical density’ (*q*_*11*_) is 0, thus attributing all changes of the optical density to the growth rate. The process variance associated with the growth rate (*q*_*22*_) was tuned using simulated data such that the filter readily tracks changes of the rate, but shows no erratic rate estimate fluctuations.

When the filter is initialized, the initial *OD*_*0*_ is the measured optical density at the first measurement. The initial geometric growth rate is 1, which translates to an hourly doubling rate of 0 ± 2.59 with the corresponding initial estimate variance *p*_*22*,*0*_ = 0.0005^2^. The initial estimate variance of the optical density *p*_*11*,*0*_ is computed by a first-order Taylor approximation propagating the intensity measurement variance *R*:

$$p_{11,k}={\left(\frac{\partial }{\partial I_{k}}\left(log_{10}\frac{I_{blank}}{I_{k}}\right)\right)}^{2}\;*\;R=R/{\left(I_{k}\;*\;ln10\right)}^{2}$$ (19)

Each time a pump thrust is triggered, the estimate variance of the optical density is reset and *p*_*11*_ is recalculated according to Eq (19) for all measurement cycles during which the pump is active and the following ten measurement cycles after the pump has been turned off. This results in a high relative weight of new OD measurements for OD estimation, but a low weight for growth rate estimation, until the OD estimate is reconverged to small uncertainties.

### Software

The firmware running on the Arduino has been coded with the integrated development environment provided by the Arduino project (https://www.arduino.cc/en/Main/Software). The graphical user interface with the Kalman filter has been programmed in Python 2.7.11 (www.python.org), and makes use of libraries contained in the Anaconda Python distribution (www.continuum.io/downloads). For coding of the graphical control elements wxPython (http://www.wxpython.org/) was used. Developed firmware and software for the graphical user interface are contained in S3 File.

3D models were designed using the free software OpenSCAD (www.openscad.org).

The board scheme and layout was generated with KiCad, which is freely available (www.kicad-pcb.org).

### Simulations

Simulations were based on a geometric growth model of actual optical densities, which are assumed to be unobserved. Using a blank intensity *I*_*blank*_ of 12,000 they were converted to light intensities and overlaid with Gaussian noise with the variance 32^2^, representing the measurement variance. Dilution events were triggered whenever the apparent optical density generated this way surpassed the current target density. Each dilution event reduced the actual density by 5%.

### Culture at different optical densities

*E*. *coli* BL21 transformed with a GFP expressing plasmid carrying an ampicillin resistance marker were used for continuous culture at different optical densities and assessment of measurement linearity. As growth medium throughout the experiment, DYT (16 g/l tryptone, 10 g/l yeast extract, 5 g/l NaCl) supplemented with 100 μg/ml ampicillin was used. Cells were inoculated from an overnight culture into fresh medium at a dilution of 1:100 and grown at 37°C for 2h. 200 μl of this culture were used to inoculate the turbidostat. Cells were grown at 37°C at increasing optical densities (0.05, 0.1, 0.2, 0.5, and 1) with cultivation for an hour at each target density once it had been reached. Unless noted otherwise, given ODs refer to turbidostat measurements. To this end, individual linear regressions of log_2_ transformed ODs were performed, with the respective slopes equaling the average doubling rates during those periods.

### Mixed culture

*E*. *coli* strains BL21 and XL1-Blue (Stratagene), both transformed with a plasmid conferring kanamycin resistance, were picked into M9 medium supplemented with 0.2% (w/v) casamino acids, 1 mM thiamine hydrochloride, and 50 μg/ml kanamycin. From respective overnight cultures, 20 ml of fresh medium were inoculated with 10 μl in case of BL21 or 400 μl for XL1-Blue. Those cultures were incubated for 5h at 37°C under shaking and then mixed 1:500 (BL21 to XL1-Blue) in respect to their optical densities at 600 nm. This mixture was used to inoculate the turbidostat to an optical density of about 0.1. The growth rate was monitored for 20h at 37°C in the supplemented M9 medium and a target OD of 0.1. For comparison, both strains were also cultured separately in the same way.

## Supporting information

S1 Fig Linearity of optical density (OD) measurements performed in the turbidostat. Eight *E*. *coli* culture samples with optical densities between 0.024 and 1.982 were taken from the turbidostat and measured in a photometer at both 600 nm (blue squares) and 650 nm (red circles) in a 1 cm path length cuvette. Linear regressions for both wavelengths up to an OD of 1.5 in the turbidostat are displayed as dashed lines with annotated slope and coefficient of determination.
(PNG)

S2 Fig Models of 3D printed parts. (A) Optics holder. (B) Tube holder. (C) Housing upper part. (D) Base plate. (E) Housing lower part. (F) Bottom cover. (G) Optics and tube holder assembled on base plate with board. (H) Fully assembled turbidostat central unit (4-pin connector to pumps not shown).
(PNG)

S3 Fig Board schematic of custom board connected to the Arduino. The two MOSFETs (U1 and U2) handle the currents driving the pumps and the stirrer motor. Furthermore, the board connects to a pair of light-to-frequency converters, the laser and a Hall sensor mounted on the board, which measures the stirrer speed.
(PDF)

S1 Table List of used commercial parts with suppliers. (DOCX)

S1 File 3D printed parts as STL-files for printing. (ZIP)

S2 File Board layout for custom Arduino shield. (ZIP)

S3 File Firmware and graphical user interface files. (ZIP)

S4 File Video clip of state estimator output for an *E*. *coli* BL21 culture, sped up by a factor of 10. (MP4)
