# Supplementary figures and images for: A user-friendly, low-cost turbidostat with versatile growth rate estimation based on an extended Kalman filter
Source: PLoS One. 2017 Jul 26;12(7):e0181923. doi: 10.1371/journal.pone.0181923 (PMC5529016; doi:10.1371/journal.pone.0181923)

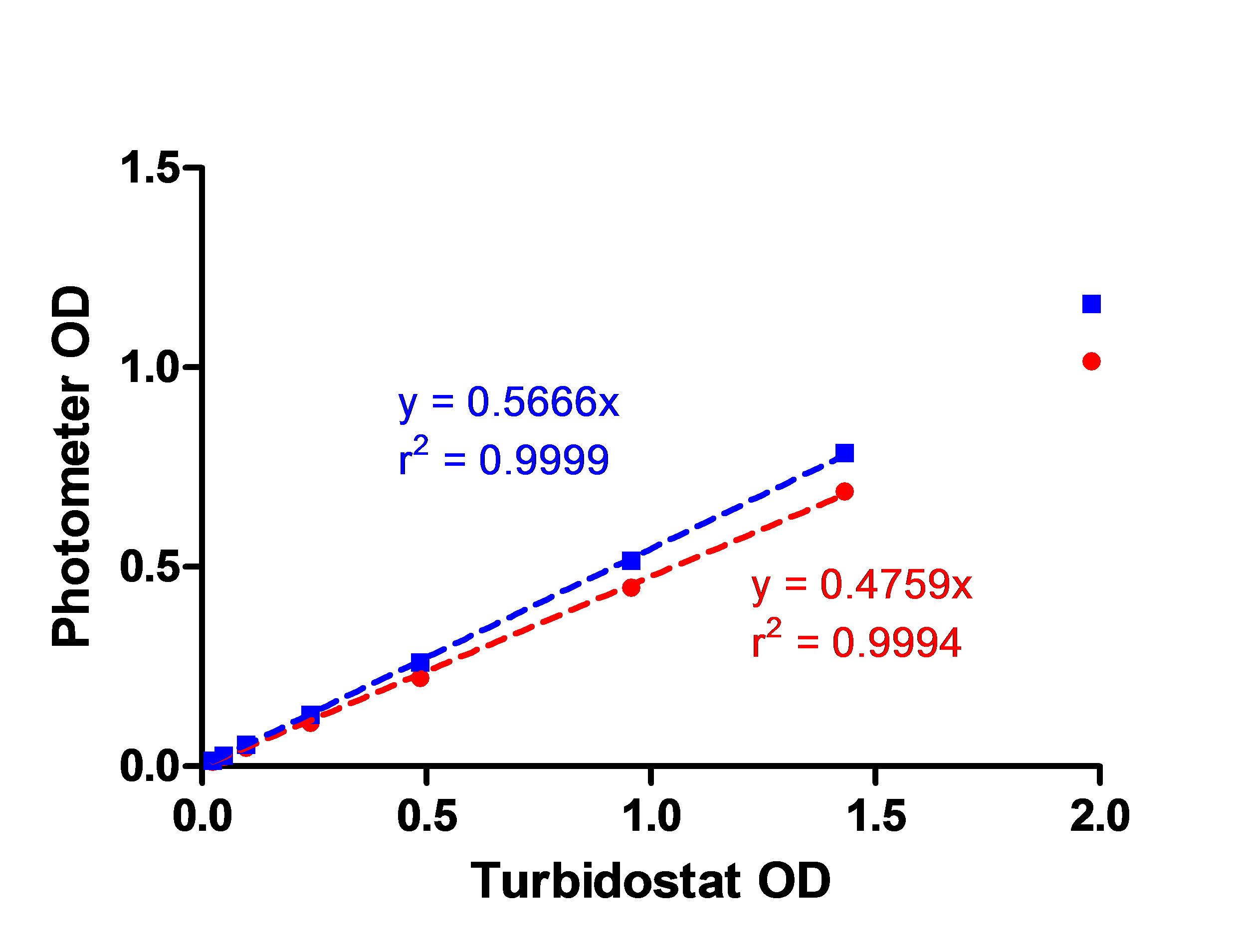

Supplement: S1 Fig — Eight E. coli culture samples with optical densities between 0.024 and 1.982 were taken from the turbidostat and measured in a photometer at both 600 nm (blue squares) and 650 nm (red circles) in a 1 cm path length cuvette. Linear regressions for both wavelengths up to an OD of 1.5 in the turbidostat are displayed as dashed lines with annotated slope and coefficient of determination. (PNG) [file pone.0181923.s001.png]

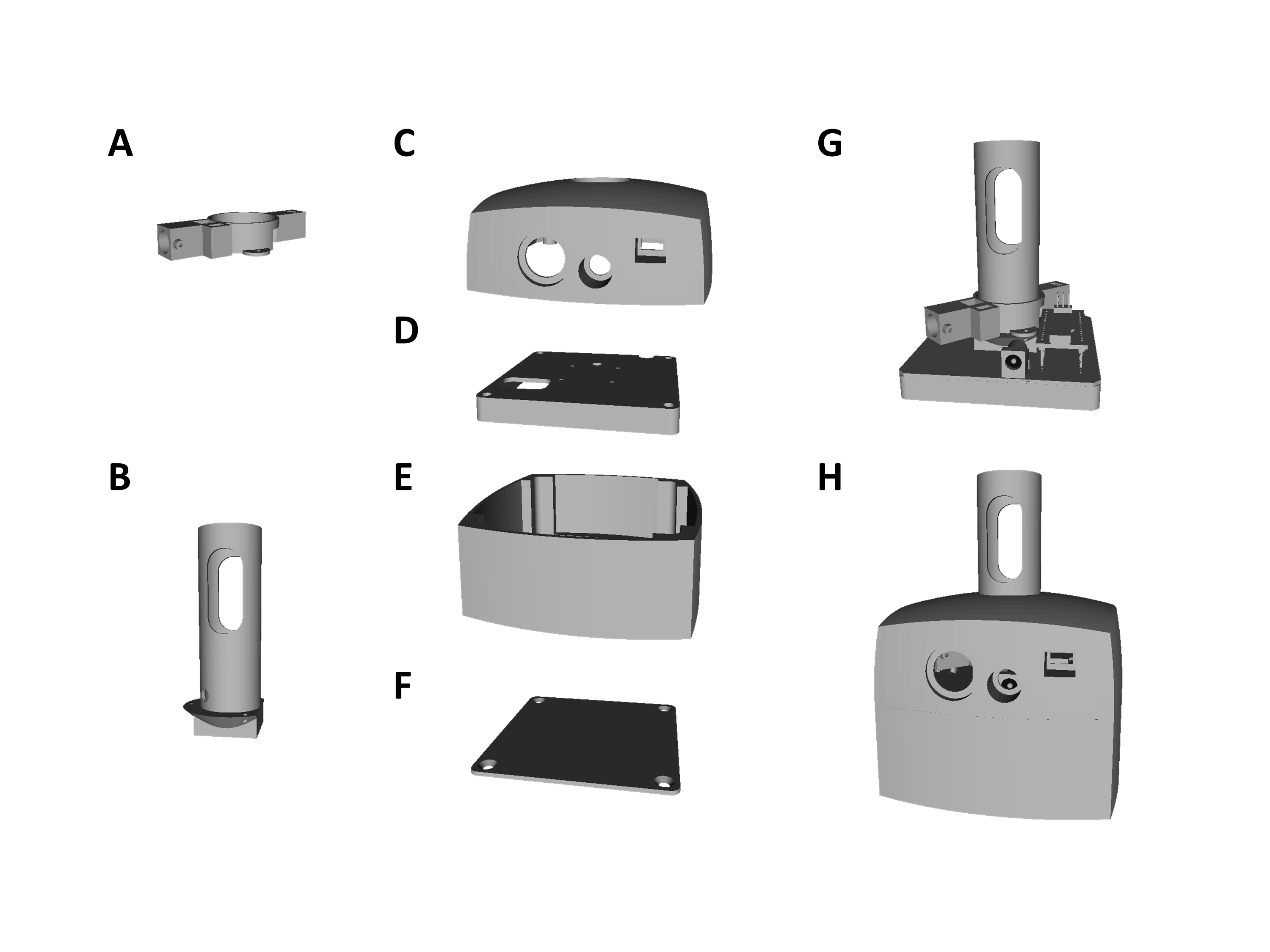

Supplement: S2 Fig — (A) Optics holder. (B) Tube holder. (C) Housing upper part. (D) Base plate. (E) Housing lower part. (F) Bottom cover. (G) Optics and tube holder assembled on base plate with board. (H) Fully assembled turbidostat central unit (4-pin connector to pumps not shown). (PNG) [file pone.0181923.s002.png]

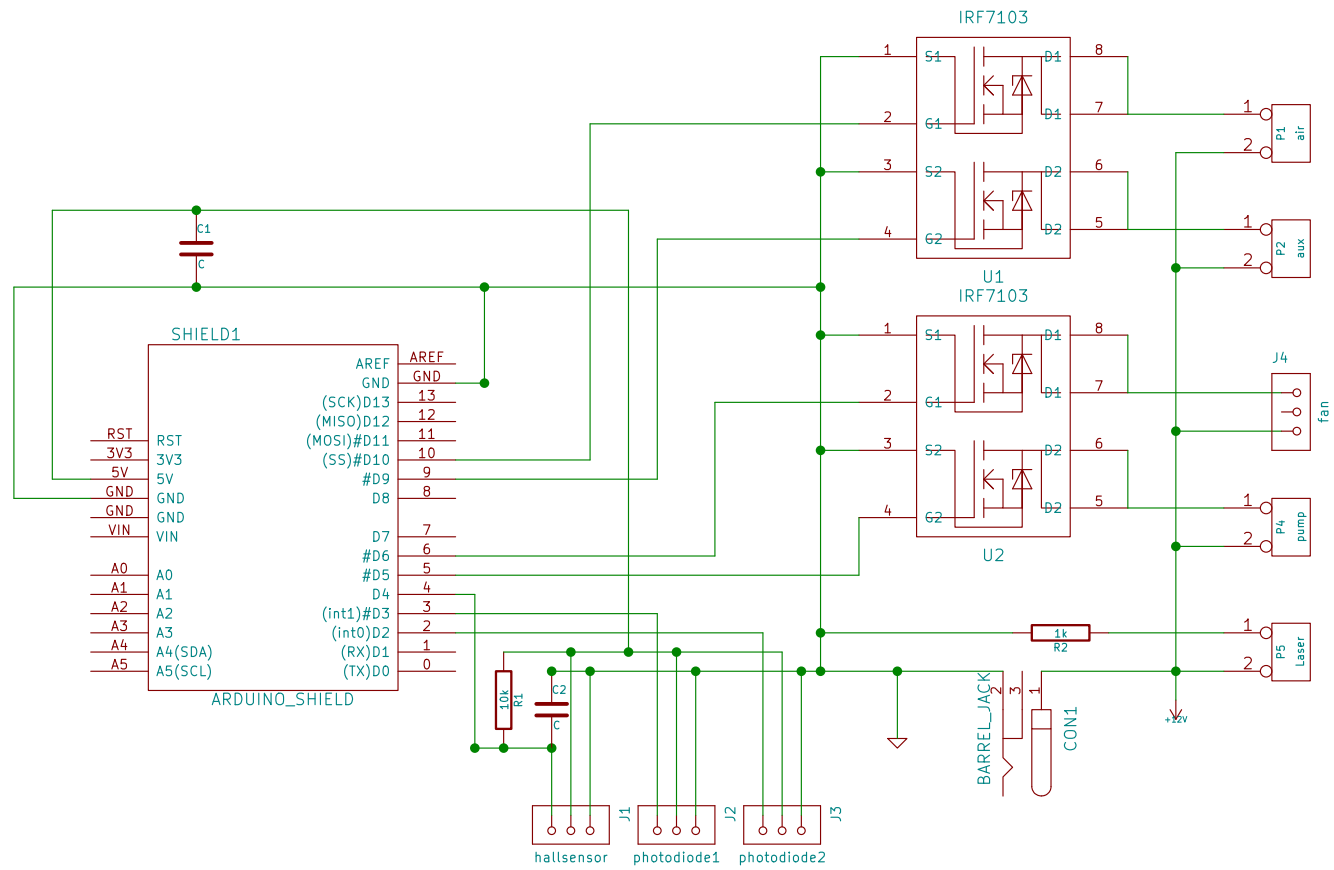

Supplement: S3 Fig — The two MOSFETs (U1 and U2) handle the currents driving the pumps and the stirrer motor. Furthermore, the board connects to a pair of light-to-frequency converters, the laser and a Hall sensor mounted on the board, which measures the stirrer speed. (PDF) [file pone.0181923.s003.pdf]

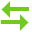

Supplement: S3 File — (ZIP) [file pone.0181923.s007.zip › Software/GUI/res/connected.png]

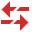

Supplement: S3 File — (ZIP) [file pone.0181923.s007.zip › Software/GUI/res/disconnected.png]

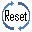

Supplement: S3 File — (ZIP) [file pone.0181923.s007.zip › Software/GUI/res/reset.png]
