# Supplementary material for: A user-friendly, low-cost turbidostat with versatile growth rate estimation based on an extended Kalman filter
Source: PLoS One. 2017 Jul 26;12(7):e0181923. doi: 10.1371/journal.pone.0181923 (PMC5529016; doi:10.1371/journal.pone.0181923)
Supplement: S1 Table — (DOCX) [file pone.0181923.s004.docx]

| Part | Supplier URL | Supplier's order number | Price per unit | Amount | total |
| --- | --- | --- | --- | --- | --- |
|  |  |  |  |  |  |
| Electronics, central unit |  |  |  |  |  |
|  |  |  |  |  |  |
| Photo-coated PCB 200x300 mm | conrad.de | 523695 | 6.86 € | 1 | 6.86 € |
| MOSFET IRF7103 SO-8 | conrad.de | 162431 | 0.50 € | 2 | 1.00 € |
| Laser module 1 mW LFD650 | conrad.de | 816476 | 7.50 € | 1 | 7.50 € |
| bar magnet 5 mm x 20 mm | conrad.de | 505782 | 1.93 € | 2 | 3.86 € |
| light-to-frequency converter TSL 235 R | conrad.de | 178993 | 3.41 € | 2 | 6.82 € |
| 12V power supply | conrad.de | 512685 | 7.99 € | 1 | 7.99 € |
| Arduino Nano | reichelt.de | ARDUINO NANO | 24.90 € | 1 | 24.90 € |
| connector for CPU fan | reichelt.de | PSS 254/3G | 0.05 € | 1 | 0.05 € |
| female connector, 4 pins | reichelt.de | M 604 | 0.48 € | 2 | 0.96 € |
| male connector, 4 pins | reichelt.de | B 604 | 0.41 € | 2 | 0.82 € |
| foil capacitor 4.7 nF | reichelt.de | MKS02-250 4,7N | 0.16 € | 1 | 0.16 € |
| foil capacitor 100 nF | reichelt.de | MKS-2-5 100N | 0.09 € | 1 | 0.09 € |
| Hall sensor | reichelt.de | TLE 4905L | 0.67 € | 1 | 0.67 € |
| 12V barrel connector | reichelt.de | HEBW 25 | 0.30 € | 1 | 0.30 € |
| Female pin header | reichelt.de | BL 1X20G 2,54 | 0.72 € | 2 | 1.44 € |
| CPU fan 80 mm as stirrer motor | reichelt.de | LÜFTER-8025 12V | 1.45 € | 1 | 1.45 € |
| long M3 screws, flat-head 50 mm | various suppliers |  | 0.30 € | 4 | 1.20 € |
| sheet metal screws 7 mm | various suppliers |  | 0.10 € | 7 | 0.70 € |
| microscope cover slip as beam splitter | various suppliers |  | 0.10 € | 1 | 0.10 € |
|  |  |  |  |  |  |
|  |  |  |  |  |  |
| Tubing system, culture vessel |  |  |  |  |  |
|  |  |  |  |  |  |
| 2x GL 45 lid with tube connectors | carlroth.com | PY86.1 | 50.58 € | 1 | 50.58 € |
| Luer-tubing connector male | carlroth.com | CT61.1 | 14.16 € | 0.3 | 4.25 € |
| Luer-tubing connector female | carlroth.com | CT62.1 | 14.16 € | 0.2 | 2.83 € |
| magnetic stirrer bar, Ø 6 mm x 15 mm | carlroth.com | AKP5.1 | 2.26 € | 1 | 2.26 € |
| injection cannulas, Ø 2 mm x 100 mm | de.vwr.com | 612-0359 | 50.10 € | 0.25 | 12.53 € |
| sterile filter 0.2 µm | de.vwr.com | 514-0061 | 33.00 € | 0.02 | 0.66 € |
| flat bottom test tube 100x25 mm | laborglasshop.de | 100103 | 12.95 € | 0.03 | 0.39 € |
| pump tubing | gemke-schlauchpumpen.com | Santoprene 4.8x8.0 mm | 10.35 € | 1 | 10.35 € |
| silicone tubing, inner Ø 5 mm, outer Ø 8 mm | various suppliers |  |  |  | 5.00 € |
| silicone tubing, inner Ø 1 mm, outer Ø 3 mm | various suppliers |  |  |  | 5.00 € |
| 2 component casting silicone | various suppliers |  |  |  | 2.00 € |
|  |  |  |  |  |  |
|  |  |  |  |  |  |
| Pump unit |  |  |  |  |  |
|  |  |  |  |  |  |
| 12V air pump | pollin.de | 330 036 | 2.50 € | 1 | 2.50 € |
| peristaltic medium pump | gemke-schlauchpumpen.com | CP82 24V 30 UPM | 45.00 € | 1 | 45.00 € |
| pump housing | conrad.de | 526033 | 8.08 € | 1 | 8.08 € |
|  |  |  |  |  |  |
| total |  |  |  |  | **218.29** € |
